# Dr. Frank E. Burdick

**Published:** 1918-02

**Authors:** 


**Dr. Frank E. Burdick, Syracuse** 1895, died at his home in Providence, R. I., Dec. 26, of heart disease, aged 46.

